# Pediatric diabetes prediction using deep learning

**DOI:** 10.1038/s41598-024-51438-4

**Published:** 2024-02-20

**Authors:** Abeer El-Sayyid El-Bashbishy, Hazem M. El-Bakry

**Affiliations:** 1 Information Systems Department, Faculty of Computer and Information Sciences, Mansoura University, Mansoura, Egypt; 2 Head of Information Systems Department, Faculty of Computer and Information Sciences, Mansoura University, Mansoura, Egypt

**Keywords:** Computational biology and bioinformatics, Diseases, Engineering

## Abstract

This study proposed a novel technique for early diabetes prediction with high accuracy. Recently, Deep Learning (DL) has been proven to be expeditious in the diagnosis of diabetes. The supported model is constructed by implementing ten hidden layers and a multitude of epochs using the Deep Neural Network (DNN)-based multi-layer perceptron (MLP) algorithm. We proceeded to meticulously fine-tune the hyperparameters within the fully automated DL architecture to optimize data preprocessing, prediction, and classification using a novel dataset of Mansoura University Children's Hospital Diabetes (MUCHD), which allowed for a comprehensive evaluation of the system’s performance. The system was validated and tested using a sample of 548 patients, each with 18 significant features. Various validation metrics were employed to ensure the reliability of the results using cross-validation approaches with various statistical measures of accuracy, F-score, precision, sensitivity, specificity, and Dice similarity coefficient. The high performance of the proposed system can help clinicians accurately diagnose diabetes, with a remarkable accuracy rate of 99.8%. According to our analysis, implementing this method results in a noteworthy increase of 0.39% in the overall system performance compared to the current state-of-the-art methods. Therefore, we recommend using this method to predict diabetes.

## Introduction

Diabetes rates exhibit an alarming annual increase, particularly when left untreated. Diabetes is a chronic pathological disease that arises due to the excessive presence of glucose in the bloodstream. Individuals with diabetes mellitus are unable to produce adequate amounts of insulin, a hormone secreted by the pancreas. Insulin plays a crucial role in regulating cellular glucose levels and is essential for energy production. Many complications may occur if diabetes remains untreated such as visual impairment, cardiology problems, dental diseases, stroke, and microvascular complications that lead to retinopathy, kidney failure, and nerve damage^1^. Diabetes management tools are essential for monitoring glucose, insulin levels, and meal ingestion. These tools include activity bands, glucose meters, continuous glucose monitoring devices, and sensor-augmented insulin pumps^2^. The primary objective of glucose management is to prevent undesired glycemia and its related events^3^. It is important to note that there are three distinct types of diabetes: type 1, type 2, and gestational. In type 1 diabetes, the pancreas produces little or no insulin. Insulin therapy is a necessary treatment for type 1 diabetes. It is usually seen in young individuals (age < 30), as well as children. Type 2 diabetes, on the other hand, is usually caused by insulin resistance and is more prevalent in older patients (age > 65 years) as well as obese patients. Gestational diabetes is hyperglycemia which occurs during pregnancy is also a concern^4^. Therefore, early detection of diabetes is critical for timely treatment and prevention of disease progression. To reduce the number of diabetes-related deaths, a useful DL technique can aid automatic disease detection. DL is a new technology that extends the machine learning (ML) technique, which is a sub-domain of artificial intelligence technology and has made significant progress in medical applications. DL uses supervised deep neural networks to perform data processing, classification, and computations of large amounts of data^5^. It allows the input of raw data and requires minimal feature engineering work on data preprocessing to learn representation automatically by exploiting the DL technique. This helps the healthcare provider detect the disease in its early stages. DNN is an ANN with deep layers. Deep layers indicate that the network has several layers: an input layer, a hidden layer, and an output layer. The number of hidden layers is greater than or equal to two connected for processing and learning from data^6^.

**Figure 1:**
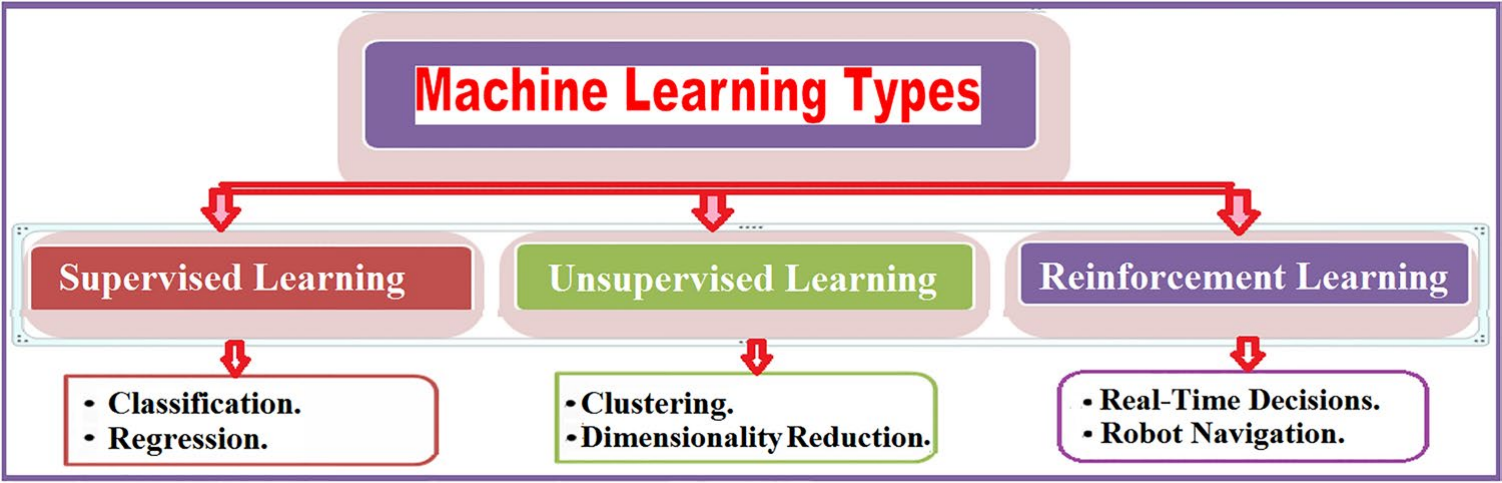
Machine Learning Types.

**Figure 2:**
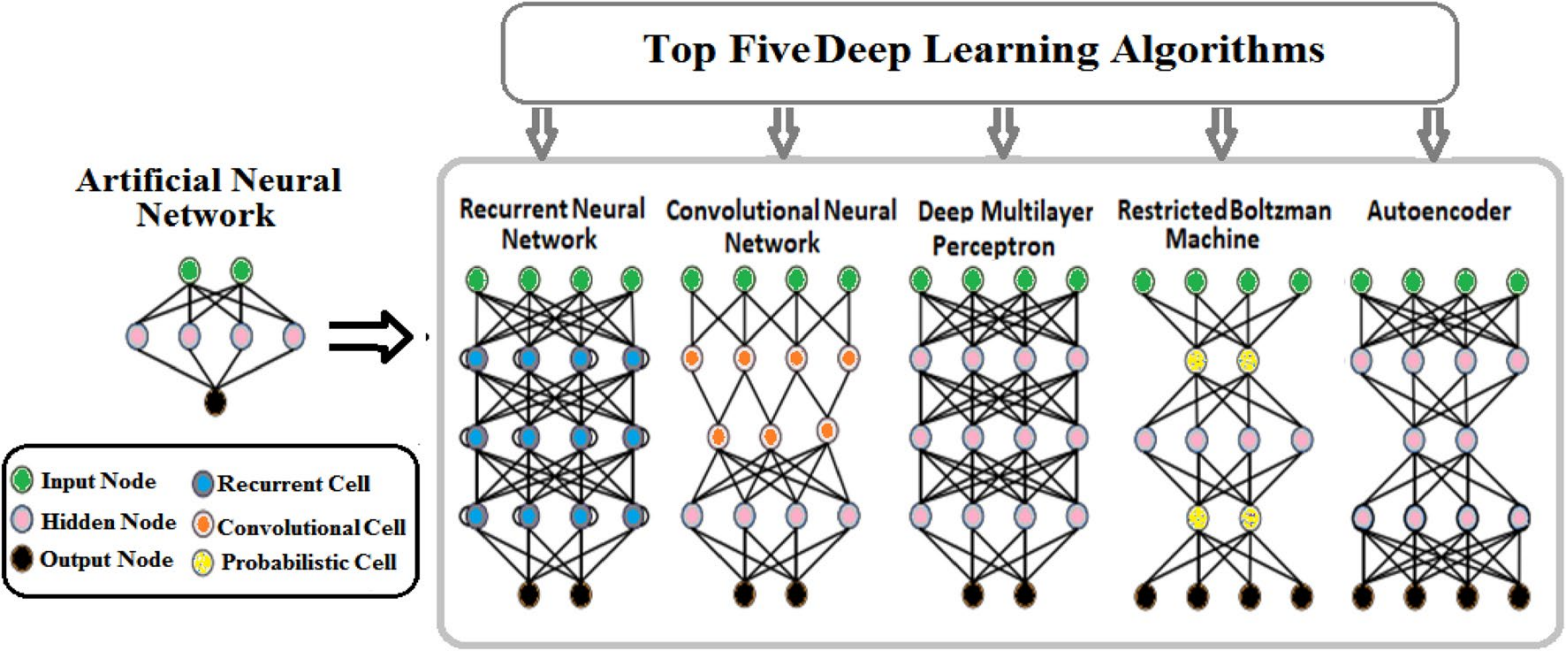
DL Algorithms.

Figure 1 shows the three ML types. These are Supervised learning (SL), Unsupervised learning (USL), and Reinforcement Learning (RL). SL uses labeled input data during iterative model optimization and backward propagation. There are many preferable SL algorithm-based DL such as Convolutional Neural Network (CNN), Multi-Layer Perceptron (MLP), and recurrent neural network (RNN) (see Figure 2). CNN can process the signals of multidimensional arrays to achieve high-performance image recognition tasks. CNN is commonly used in image recognition and computer vision. MLP is a feed-forward neural network associated with a set of bias scalars, weights, and activation functions. Common tasks of SL are classification and regression. RNN captures temporal features by preserving information from the time series, as seen in speech recognition, and natural language processing. In USL predefined labels or classes of inputs are not required for training the model. Instead, the algorithm aims to infer hidden structures and representations from the input datasets without supervision. Common tasks of USL are cluster analysis and dimension reduction. Restricted Boltzmann Machines (RBMs) and autoencoders are the two basic USL architectures. RBMs map the representations by estimating a probabilistic distribution over the input data. Autoencoders are used when the number of output units in the output layer is the same as the number of input units in the input layer, and RL is a ML algorithm inspired by how humans learn from trial and error. The agent interacts with a high-dimensional environment and learns from their experiences to obtain the optimal outcome, which leads to obtaining the maximum reward of feedback to the algorithm to learn from and improve future results. The common tasks in RL are real-time decisions and robot navigation^7^.

The rest of this paper is organized as follows: section related work discusses related work reported in literature, Sect. 3 the proposed architecture and its corresponding features describes a detailed explanation of the proposed model. In Sect. 4, the results and discussion are presented. Conclusion and future work are presented in Sect. 5.

## Related work

Many studies use four DL algorithms CNN, Deep Belief Network (DBN), Deep Neural Network (DNN), and MLP on the Pima Indian diabetes (PID) dataset, which consists of nine attributes, and 768 records describing female patients^8^. Wee et al.^9^, increased the dataset size using the Variational Auto Encoder (VAE) and a Sparse Auto Encoder (SAE) to increase the feature number. The CNN algorithm was used to generate a feature map that clustered and summarized the information in the dataset. The feature map is then passed down to the pooling layer. This assists in decreasing the computation power and time. The activation functions SoftMax and Sigmoid were utilized. The DBN algorithm is based on multiple RBMs to determine the probability distribution of a dataset. The DNN is the result of combining multiple hidden layers and MLP is combined with augmentation. The CNN with the SAE achieved a high accuracy of 98.1 among all state-of-the-art methods.

Naseem et al.^10^ provide an Internet of Things (IoT) platform in a healthcare system based on ML and DL. IoT is concerned with information extraction, and it transmits real-time data from the human body such as diabetes sensors. They used a Support Vector Machine (SVM), Logistic Regression (LR), Artificial Neural Network (ANN), CNN, RNN, and Long Short-Term Memory (LSTM) algorithms with two activation functions namely Sigmoid and a Rectified Linear unit (ReLU) on the PID dataset. The RNN achieved the best accuracy of 81% compared with the other classifiers.

Khanam and Foo^11^ used ANN, Data mining, and ML algorithms to predict diabetes on the PID dataset. The model works well with LR and SVM with accuracies of 78.8571% and 78.2857%, respectively, for diabetes prediction. The ANN model with various epochs and two hidden layers provided a high accuracy of 88.6% using the ReLU activation function.

In addition, García-Ordás et al.^12^ used autoencoder, MLP, and CNN algorithms to deal with unbalanced datasets. They performed data augmentation using the VAE. VAE is part of an autoencoder technique that tries to learn a deep representation of the data by compressing the features. They perform feature augmentation using SAE. The latent space layer has more neurons than the input and output layers. They achieved an accuracy of 92.31% when the CNN classifier was trained jointly with the SAE to feature augmentation using a sigmoid function over a well-balanced PID dataset.

Kumar et al.^13^ applied the Multi-Layer Feed Forward Neural Networks (MLFFNNs) with a backpropagation algorithm to classify diabetes on the PID datasets. They use various activation functions such as scaled exponential linear units and exponential linear units (ELU). The ELU controls the saturation of the negative inputs. this helps in accelerating the learning process. In addition, they compared the performance of two ML algorithms: Naive Bayes (NB) and Random Forest (RF). The result of their study demonstrated a classification accuracy of 84.17%.

Krishnan^14^ presented an automatic classification of diabetes disease using MLP and SVM classifiers to achieve the best therapeutic management on the PID dataset. The MLP classifier fine-tuned the hyper-parameters to minimize the loss function and optimize the supported model as much as possible, such as Adams updater with a learning rate of 0.001, activation function as Gaussian Radial Basis Function Network, Batch processing with seed of 0, optimizer as stochastic gradient descent, and 10-fold cross-validation. The results indicate that The MLP with the SVM classifier achieved a high classification accuracy of 77.47%. in comparison.

Zhou, Myrzashova, and Zheng^15^ constructed a model to classify diabetes and diabetes types. They added a normalization layer called dropout regularization to reduce the overfitting problem and fine-tuned the hyperparameters with the binary cross-entropy loss function to enhance the network efficiency. The Ada Delta parameter restricts the window of the accumulated past gradients to a fixed size. The best training accuracy for the diabetes type was 94.02174% using the diabetes type dataset from the Data World repository, and for the diabetes prediction was 99.4112% using the PID dataset.

According to previous studies, all the patients in the most popular PID dataset are female with the number of pregnancies and are at least 21 years old. The PID dataset has many missing values that require hard data preprocessing to avoid inaccurate prediction. If the missing data are eliminated, the dataset will be small. This results in inaccurate predictions and a notable problem with the accuracy. We strive to elevate the accuracy to a higher level. Additionally, we must identify further significant features that can contribute to precise diabetes prediction. In addition, the study includes a small age group of patients features that must be taken into consideration.

## Methods

Disease classification assigns patients’ information to binary categories based on their medical attributes. This was accomplished by building a model using a train-and-test cycle. It is important to note that this technique is considered a supervised classification technique. A set of predefined labeled attributes is provided as a training set to aid in the classification of new, unknown datasets. However, the size of the dataset can present a significant challenge owing to the high dimensionality of irrelevant attributes, resulting in poor classification performance. This problem can be handled through a preprocessing step by building an aperture classification algorithm designed using statistics and control theory to analyze and retrieve knowledge from experience.

Following the proposed model, it was partitioned into three distinct layers. The input layer is responsible for receiving seventeen raw input data features from the domain without any prior computation. The input features are then subjected to a DNN architecture that guarantees ten hidden layers. The input features are assigned initialized weight values ranging from (0 to 1). All neurons in the hidden layers are attached to both the previous and next layers. At the hidden layers, each node provides an abstraction to the NN with all feature computations. The result is then transferred to the output layer, which assimilates the information learned through the hidden layer and provides the binary classification output. Parameters such as weights and biases, are initially randomized and subsequently adjusted to optimize the prediction model. The proposed model will yield greater efficiency in the field of medical disease classification field. The model has been presented utilizing the DNN technique, with fine-tuned hyperparameters that assist in building a robust model for the classification of diabetes disease, based on the medical records of the MUCHD dataset.

**Figure 3:**
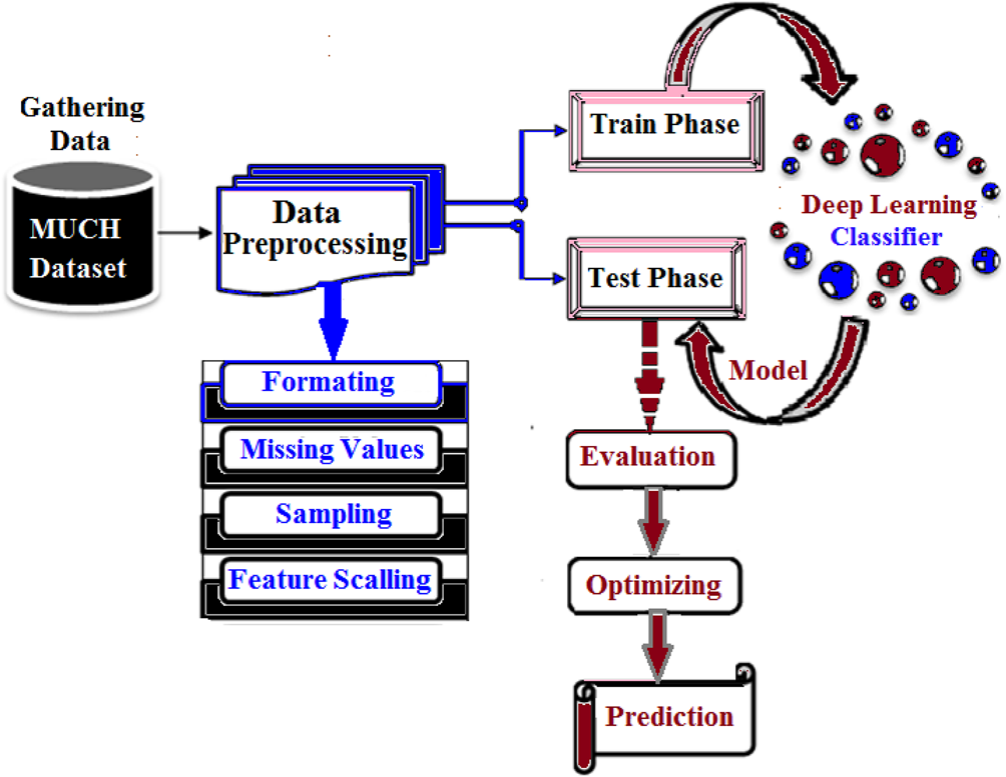
Diabetes System Architecture.

The model under consideration comprises a sequence of phases, as presented in Figure 3. The system includes a data collection phase, a data preprocessing and classification phase, a train/test phase, an evaluation phase, an optimizing phase, and a prediction phase. The data is sourced from the MUCHD dataset. During the preprocessing phase, it is of utmost importance to enhance the features of missing values and remove insignificant features to avoid misleading results. Subsequently, the features are scaled to a normalized scale. The normalized preprocessed features are then fed to the classifier. The model learns from the trained labeled data and tests its performance using the unlabeled test data. Following The validation phase, the classifier performance is evaluated, followed by the optimizing phase, and ultimately culminating in the prediction phase.

We present below the six phases of the proposed system in detail:

### Data collection phase

The quality of the data quantity is a crucial factor that cannot be overlooked. The model will be powerful when picking the right data. The reliability of data plays a significant role in all model classification phases, which leads to useful predictions. During this phase, careful consideration is given to the selection of data features and the total number of samples. Assumptions are made regarding the most related data to diabetes. Hence, good data leads to good performance and a successful model.

### Dataset availability

The proposed model employs a MUCHD dataset of pediatric patients, encompassing individuals ranging from one year to nineteen years old both male and female. This dataset is obtained from the Mansoura University Children's Hospital repository system, Medicine Faculty, and Dakahlia Governorate of Egypt. Data is gathered from patient medical records through examination, laboratory tests, and admission notes. The MUCHD dataset comprises 548 records classified into two distinct classes: diabetes and non-diabetes (healthy). These records are associated with 18 attributes, which include Age, Sex, Duration, Cholesterol, Creatinine, Acetone, Glycated Haemoglobin (HbA1c), Insulin level, Post Prandial C-Peptide (PCPeptide), Fast C-Peptide (FCPeptide), two hours Post Prandial Blood Glucose (PBGlucose), Fast Blood Glucose (FBGlucose), Random Blood Glucose (RBGlucose), Blood Gases include Blood Acidosis(PH), Bicarbonate(HCO3), Sodium (Na), Potassium (K) and the output target feature named Diagnosis. The ‘Diagnosis' output attribute consists of binary values. It has one value of either 1, indicating the presence of diabetes in 397 patients, or 0 indicating non-diabetes patients in 151 patients. Table 1 presents the MUCHD dataset of 18 attributes along with their comprehensive descriptions. The diagnosis attribute is considered the dependent output variable, whereas the remaining 17 attributes are regarded as independent input features.

**Table 1:** The Input Attributes of the MUCHD Dataset.

Attribute	Description
Age	Age (Years)
Sex	Gender
Duration	Period time of diabetes symptoms per week
Cholesterol	Cholesterol
Creatinine	Creatinine
Acetone	Acetone
HbA1c	Hemoglobin(A1c)
Insulin	Insulin Level
PCPeptide	Post Prandial C-Peptide
FCPeptide	Fast C-Peptide
PBGlucose	Post-Prandial Blood Glucose
FBGlucose	Fast Blood Glucose
RBGlucose	Random Blood Glucose
PH	Blood Acidosis of Blood Gases
HCO3	Bicarbonate of Blood Gases
Na	The Sodium of Blood Gases
K	Potassium of Blood Gases
Diagnosis	Diagnosis of Diabetes is 1 for a positive test and 0 for a negative test

### Data pre-processing and classification phase

This step is performed using DNN. This phase is used to ensure that the input data is presented in a clear and organized format. The primary advantage of feature extraction is its ability to identify the most effective features for the model classifier to learn the representation^16^. certain errors may arise due to human mistakes during the data collection phase, resulting in labeling errors.

#### Formatting

The dataset may not be in the correct format such as a database or CSV file. To address this, we meticulously prepared the MUCHD dataset in CSV file format.

#### Missing* values*

**Figure 4:**
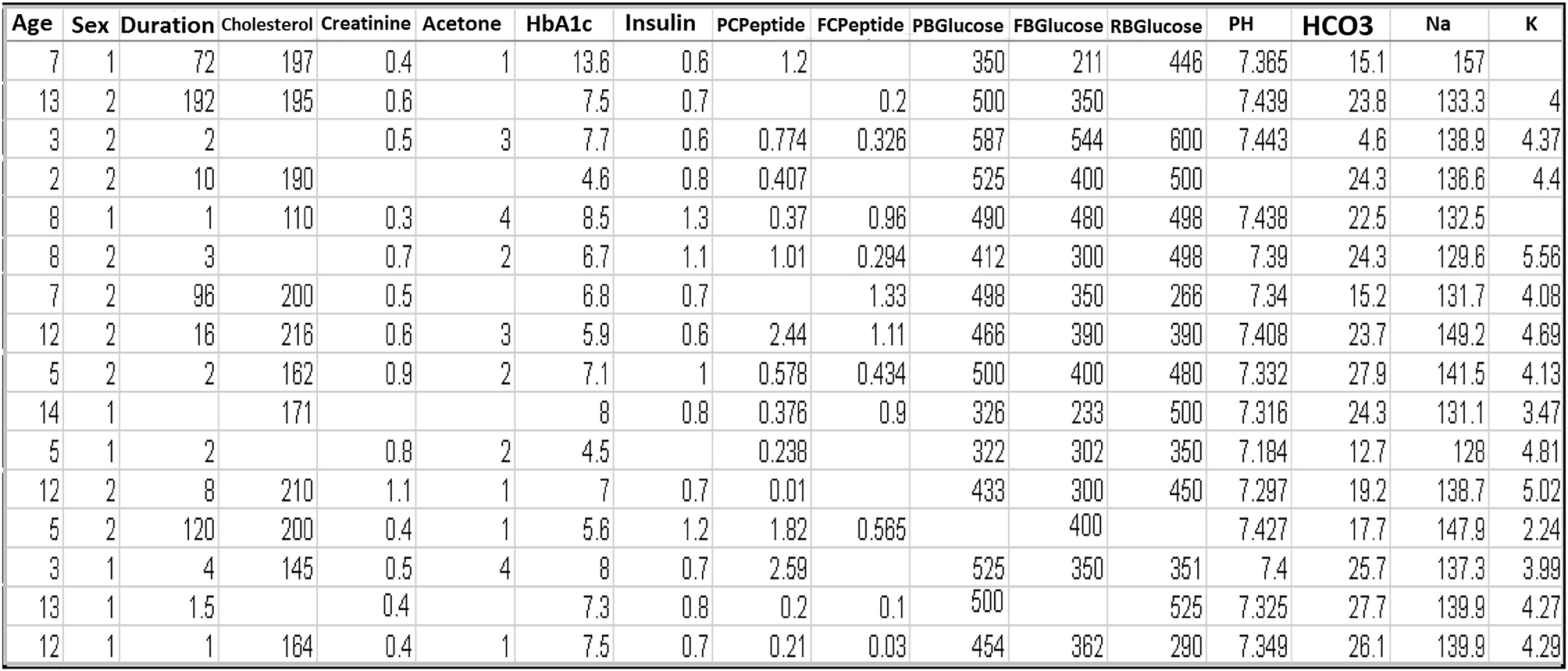
Feature Attribute Missing Value.

Dealing with noisy missing values poses a significant challenge when gathering data for DL techniques that extremely land with a perfect dataset, which will probably take a significant amount of time. Missing data samples often arise from errors in data collection as a blank space for diagnostic features that are not applicable^20^. Missing values are typically denoted as Nan or null indicators. It is necessary to delete redundant rows and columns. Consequently, two approaches are recommended to address this problem. The first involves eliminating the samples of the missing values, but it is risky to delete relevant information. The second method is to impute the missing values by replacing them with the mean value for each input feature. In Figure 4, the missing values appear as white blank.

The feature selection method provides the highest correlated values, reduces execution time, and avoids data overfitting^17^. This leads to improved performance efficiency and reduces computational requirements. We investigate the MUCHD dataset using Pearson’s correlation method applied to Python programming language and libraries (Sklearn, Tensorflow, and Keras) that have been used in data preprocessing and model implementation processes. The coefficient values remain in the range between 0 and 1. A value below 0.5 is indicative of a weak correlation, while a value above 0.5 indicates a strong correlation. A value of zero, on the other hand, signifies no correlation. Irrelevant or outlier attributes are removed from the dataset. The correlation between input/output attributes of the MUCHD dataset is indicated in Table 2. Table 3 provides a comprehensive overview of the most significant relevant features namely HbA1c, Insulin, PCPeptide, FCPeptide, PBGlucose, FBGlucose, and RBGlucose. The number of missing values for the most significant features is calculated.

**Table 2:** The Correlation Between Input/output Attributes.

Attributes	Correlation Coefficient
Age	0.07
Sex	0.059
Duration	0.24
Cholesterol	0.12
Creatinine	0.22
Acetone	0.24
HbA1c	0.71
Insulin	0.61
PCPeptide	0.37
FCPeptide	0.31
PBGlucose	0.86
FBGlucose	0.82
RBGlucose	0.73
PH	0.23
HCO3	0.16
Na	0.16
K	0.18

**Table 3:** The Number of Missing Values of the MUCHD Dataset.

Attributes	Missing values
Age	0
Sex	0
Duration	0
Cholesterol	0
Creatinine	0
Acetone	0
HbA1c	194
Insulin	76
PCPeptide	156
FCPeptide	145
PBGlucose	66
FBGlucose	78
RBGlucose	61
PH	0
HCO3	0
Na	0
K	0
Diagnosis	0

#### Sampling

Sometimes you may have too much data than you require. This can result in increased computational and memory requirements. We consider an appropriate number of samples, which will speed up the processing steps involved in exploring and prototyping the solution. The size of the data samples is determined according to the requirements for faster convergence and reduction of the disk space.

#### Feature* scaling*

In the preprocessing phase, it is crucial to take a specific step. The majority of DNN algorithms perform significantly better when dealing with features that are on the same scale^21^. The features are implemented to reduce uncertainty, incorrect results, and cost/processing time. One effective method for achieving this is feature scaling, which involves forming the smallest value of any feature to 0.0 and the largest value to 1.0. There are two common feature scaling techniques. The first is normalization, which rescales features to a range between 0.0 and 1.0. The second technique is standardization, which involves centering the field at a mean of 0.0 and a standard deviation of 1.0. The columns feature has the same parameters as a standard normal distribution, that is, zero mean and unit variance. This makes it much easier for the learning algorithms to learn the weights of the parameters as well as valuable information on the outliers, thereby rendering the algorithms less susceptible to their influence. Generally, when we input data into DNN algorithms, it is customary to manipulate the input data in such a way that the values are adjusted to a balanced scale. The missing values are substituted with the corresponding mean value after normalization. Normalization of data is a crucial step to ensure that the model can be generalized appropriately. Scaling the data is achieved through the utilization of Eq. (1) ^18^.

1 $$Z=\frac{{X}_{i}-\mu }{\sigma }$$

where Xi data is rescaled into Z with μ = 0 and σ = 1.

The statistical computations of the mean, standard deviation (std), minimum (min), and maximum (max) values for all attributes before and after the normalization process are shown in Tables 4 and 5.

**Table 4:** The Statistical Computations Before Normalization.

Attributes	Mean	Std	Min	Max
Age	9.19	4.07	1	19
Sex	1.48	0.50	1	2
Duration	57.41	90.02	0.3	768
Cholesterol	167.22	44.47	46	518
Creatinine	0.69	0.30	0.2	5.3
Acetone	2.39	0.52	1	6.1
HbA1c	7.60	1.68	0	14.6
Insulin	2.01	2.78	0.1	65
PCPeptide	1.09	0.33	0	5
FCPeptide	0.80	0.15	0.1	3
PBGlucose	277.09	95.60	80	640
FBGlucose	228.29	86.49	70	600
RBGlucose	414.71	75.10	39	750
PH	7.32	0.23	2.3	8
HCO3	20.80	5.11	2.2	44
Na	137.59	6.83	124.8	235.4
K	3.91	0.52	0.4	6.8

**Table 5:** The Statistical Computations after Normalization:

Attributes	Mean	Std	Min	Max
Age	9.19	4.07	1	19
Sex	1.478	0.50	1	2
Duration	37.62	94.07	0	768
Cholesterol	102.84	92.80	0	518
Creatinine	0.44	0.45	0	5.3
Acetone	0.65	1.18	0	6.1
HbA1c	7.59	1.67	0.038	14.6
Insulin	2.04	2.78	0.05	65
PCPeptide	0.14	0.48	0	5
FCPeptide	0.05	0.24	0	3
PBGlucose	277.05	95.60	80	640
FBGlucose	228.27	86.49	70	600
RBGlucose	414.73	75.10	39	750
PH	4.59	3.56	0	8
HCO3	12.75	11.35	0	44
Na	77.83	68.60	0	235.4
K	2.039	2.03	0	6.8

### Train/Test phase

The dataset is split into three subsets a training set, a validation set, and a test set, which learns only from the training data, while the validation set is employed for development by fine-tuning the hyperparameters. Finally, the test set to evaluate its performance serves as the ultimate benchmark, allowing us to assess the model’s effectiveness in real-world scenarios. We divide the MUCHD dataset into smaller batches with 70% of the data allocated for training and validation purposes, and 30% reserved for testing data. We then feed these beaches into the DNN technique.

### Train algorithm

The first input layer takes the seventeen input features from the MUCHD dataset. The combination of the inputs, weights, and bias are supplied to the activation function as ReLU and Sigmoid with ten hidden layers which are passed further to the next layer. The ReLU function is efficient in computation and scale-invariant. The Sigmoid function is used to fix the data into the range [0,1] for implementation. The DNN algorithm uses a backpropagation technique for classification. The DNN must return through its layers, update the weights to improve itself and minimize the cost function back to the input. The hyperparameters are then fine-tuned. The assigned weight and bias values are updated to reduce the loss function. The Loss function measures how far the predicted output is from the actual output, which determines whether to decrease or increase the weights and bias. Compute the gradient descent which adjusts the parameters by moving to a flat region. the gradient/derivative of the cost function determines whether the weights and bias decrease or increase compared to the optimal weight value. This process is repeated until the proposed system approaches the perfect predicted output as shown in Algorithm 1. MLP is a feedforward neural networks that have various layers of perceptron. In MLP each linear combination is propagated to the next layer based on the result of their computation. the perceptron can only provide outputs in the form of 0 and 1 which will be effective in the binary classification of diabetes. The predicted output is compared with the desired output for the given input. This led to an error that we wish to minimize see Algorithm 1 and Table 6.

**Algorithm 1:**
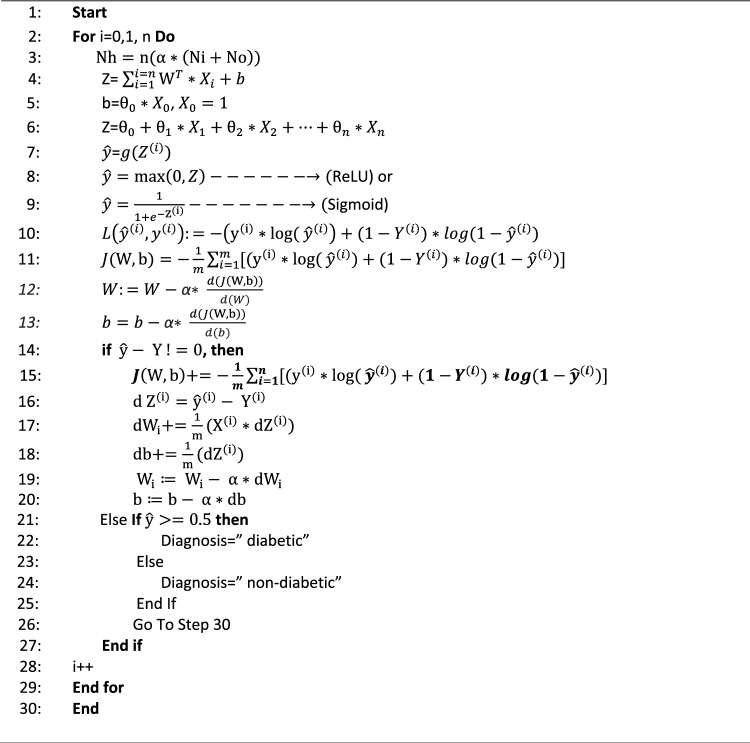
The DNN Algorithm.

**Table 6:** Notations of Algorithm 1 and their descriptions.

Notation	Description
X_1_, X_2_, X_3_,…., X_17_	The seventeen input features
${{\text{N}}}_{{\text{h}}}$	The number of neurons in the hidden layers
m	The total number of samples in the MUCHD dataset
α	An arbitrary scaling factor
N_i_	The number of neurons in the input layer
N_o_	The number of neurons in the output layer
W	Weights
$\uptheta$_0_, $\uptheta$_1_, …, $\uptheta$_17_	The eighteen input weights
Z	A linear transfer function
b	bias node
T	Transpose
i	A counter
n	The total number of input features
g	The activation function
$\widehat{y}$	The predicted output
ReLU	Rectified Linear unit
$\sigma$	the Sigmoid function
$L\left({\widehat{y}}^{\left(i\right)},{y}^{\left(i\right)}\right)$	Loss Function
Y	Actual output
$J(\uptheta ,{\text{b}})$	Cost Function
${dW}_{i}+$	Accumulative weight
$db+$	Accumulative bias

### The construction of a DNN

**Figure 5:**
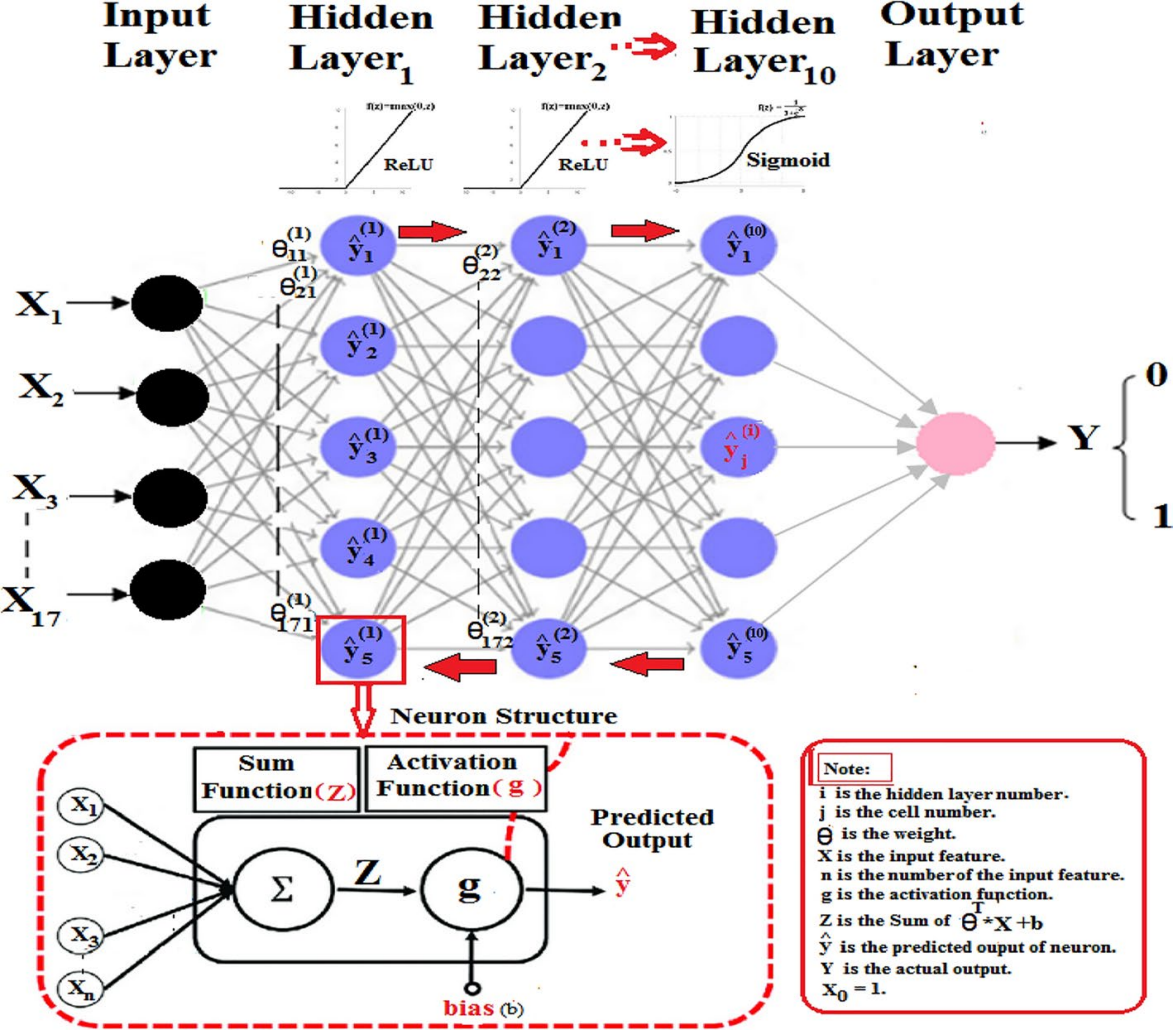
DNN architecture.

The Construction of DNN Algorithms and architecture is explained in Figure 5.

### Input layer

Input the seventeen input features [X_1_, X_2_, X_3_,...., X_17_] into the input layer. Typically, the input values are scaled to fall values between 0 and 1.

### Hidden layer

The maximum number of hidden neurons that can be used without causing overfitting using Eq. (2) ^19^.

2 $${{\text{N}}}_{{\text{h}}}={\text{m}}(\mathrm{\alpha }*\left({{\text{N}}}_{{\text{i}}}+{{\text{N}}}_{{\text{o}}}\right))$$

$$\mathrm{Nh }= 548 / (4*(17+1) =7.61 \sim 8\mathrm{ neurons}$$

In a feedforward neural network, the number of neurons in the hidden layers is denoted by N_h_ while the total number of samples in the MUCHD dataset is represented by m, an arbitrary scaling factor, α, typically falls between 2 to 10, the input layer is denoted by N_i_, and the output layer by N_o_.

**Figure 6:**
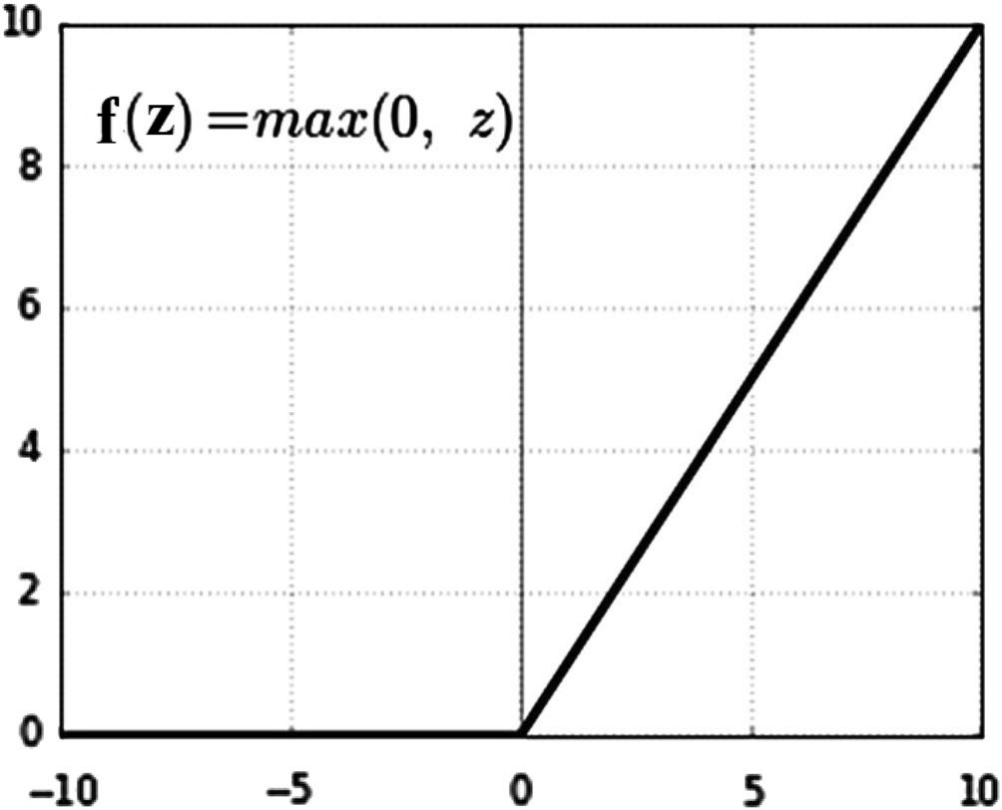
ReLU Function.

**Figure 7:**
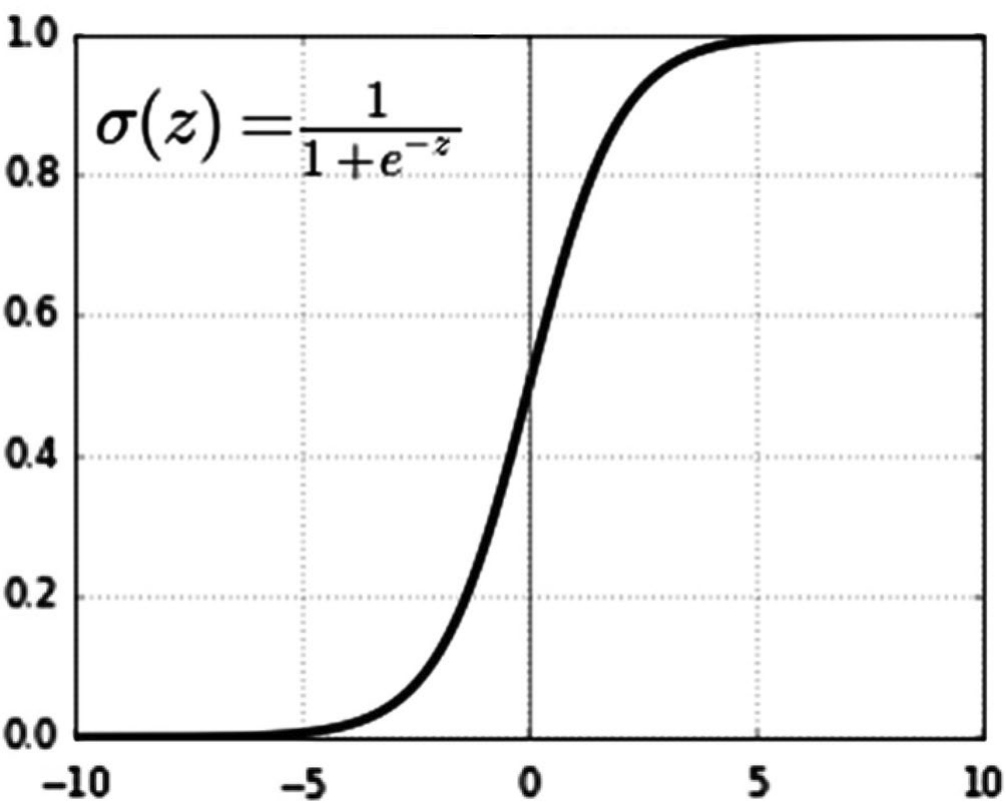
Sigmoid Function.

- Initialize the trained weights (W) parameters of the model, random real numbers between 0 and 1 are assigned to each parameter [$\uptheta$_0_, $\uptheta$_1_, …, $\uptheta$_17_].
- Let the bias node be (b).
- Let T be the transpose of weight.
- Creates a linear transfer function (Z) out of all the inputs^20^.
- Let a counter (i) be used to track the input features and n is the total number of input features. During the feedforward propagation, this process is repeated from i = 1 to n using Eqs. (3)–(6).
  3 $${\text{Z}}= \sum_{i=1}^{i=n}{{{\text{W}}}^{T}*X}_{i}+b$$
  4 $${\text{Z}}={{\uptheta }_{1}*X}_{1}+{{\uptheta }_{2}*X}_{2}+\dots +{{\uptheta }_{n}*X}_{n}+b$$
- Replace the bias factor (b) with the elements ${\uptheta }_{0}*{X}_{0}$.
  5 $${\text{Z}} = \theta_{0} * X_{0} + \theta_{1} * X_{1} + \theta_{2} * X_{2} + \cdots + \theta_{17} * X_{17}$$
- Let $${X}_{0}=1.$$
  6 $$Z = \theta_{o} + \theta_{1} * X_{1} + \theta_{2} * X_{2} + \cdots + \theta_{n} * X_{n}$$
- Apply the activation function (g). We use ReLU and Sigmoid functions to take the input from a preceding linear node (Z).
- Calculate the predicted output ($\widehat{{\varvec{y}}}$) using Eq. (7).
  7 $$\widehat{{\varvec{y}}}={g(Z}^{(i)})$$
- Apply the activation function ReLU as indicated in Fig. 6, and compute $\widehat{{\varvec{y}}}$ using Eq. (8).
  8 $$\widehat{y}=\left({Z}^{\left(i\right)}\right)={\text{max}}\left(0,\mathrm{ Z}\right)=\frac{Z+|Z|}{2}=\left\{\begin{array}{c}Z\,if\,Z>0,\\ 0\,otherwize\end{array}\right.\left\{\begin{array}{c}1\, if\, Z>0,\\ 0\, if\, Z<0\end{array}\right.$$
- Then, apply the Sigmoid function (${\varvec{\sigma}}$) as indicated in Fig. 7, and compute $\widehat{{\varvec{y}}}$ using Eq. (9).
  9 $$\widehat{y}=\sigma \left({Z}^{\left(i\right)}\right)=\frac{1}{1+{e}^{{-{\text{Z}}}^{({\text{i}})}}}$$
- The predicted output ($\widehat{{\varvec{y}}})$ of a neuron in a layer is sent as input to all neurons in the next layer as input.
- Let Y be the actual output and α be the step increment.

### Output layer

**Figure 8:**
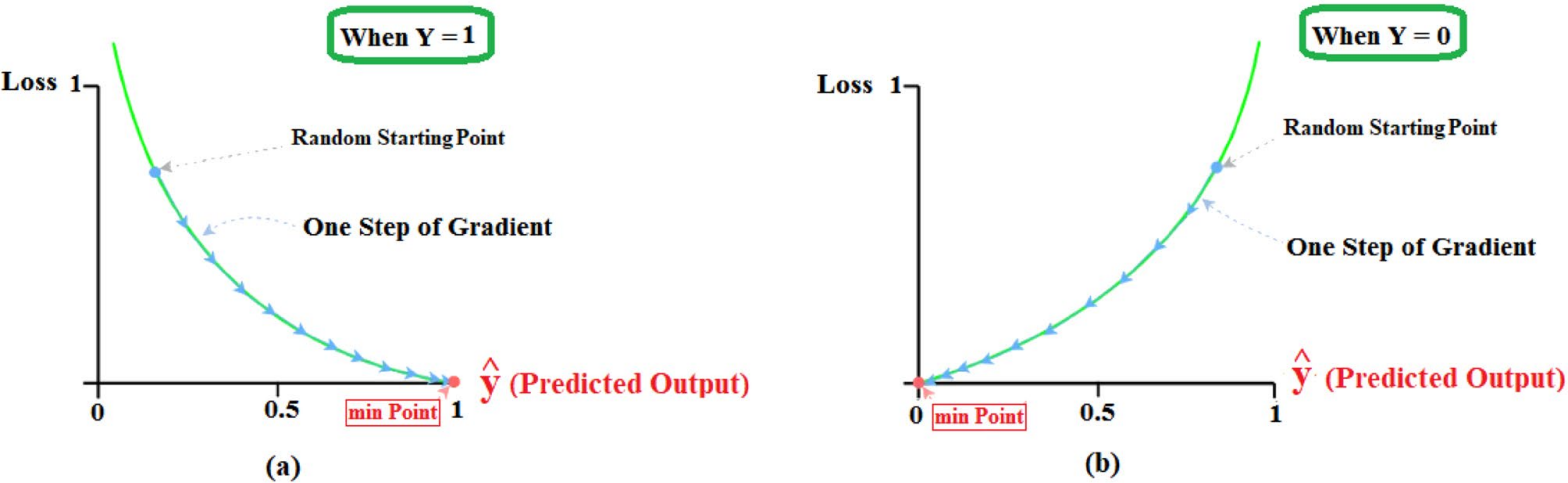
Gradient descent of Random Starting point.

- Compute Loss Function (${\varvec{L}}\left({\widehat{{\varvec{y}}}}^{\left({\varvec{i}}\right)},{{\varvec{y}}}^{\left({\varvec{i}}\right)}\right))$ using Eq. (10).
  10 $$L\left({\widehat{y}}^{\left(i\right)},{y}^{\left(i\right)}\right):=-\left({{{\text{y}}}^{\left({\text{i}}\right)}*{\text{log}}(}_{ }{\widehat{y}}^{\left(i\right)}\right)+(1-{Y}^{\left(i\right)})*log(1-{\widehat{y}}^{\left(i\right)})$$
- Compute Cost Function ${\varvec{J}}\left({\varvec{\uptheta}},\mathbf{b}\right)$ using Eq. (11).
  11 $$J({\text{W}},{\text{b}})=-\frac{1}{m}\sum_{i=1}^{n}[({{{\text{y}}}^{\left({\text{i}}\right)}*{\text{log}}(}_{ }{\widehat{y}}^{\left(i\right)})+(1-{Y}^{\left(i\right)})*log(1-{\widehat{y}}^{\left(i\right)})]$$
- Compute gradient descent as shown in Fig. 8.
- Perform regularization by Selecting the most significant feature, changing the ${\text{W}}$ value, or adding a regularization factor ($\boldsymbol{\alpha }).$
- Apply *alpha (α)* to control the step size down the Loss curve using Eq. (12–13).
  12 $$W: = W - \alpha * \frac{{d\left( {J\left( {W,b} \right)} \right)}}{d\left( W \right)}$$
  13 $$b=b-\alpha * \frac{d(J\left({\text{W}},{\text{b}}\right))}{d(b)}$$

if $\widehat{{\varvec{y}}}- {\varvec{Y}}\ne 0$, then start the backward propagation process.

- In the backpropagation process, we track from output to the input to obtain the optimal values of weights $({\text{W}})$ and bias using Eq. (14).
- Let m be the number of samples.
  14 $$J\left({\text{W}},{\text{b}}\right)+=-\frac{1}{m}\sum_{i=1}^{n}[({{{\text{y}}}^{\left({\text{i}}\right)}*{\text{log}}(}_{ }{\widehat{y}}^{\left(i\right)})+(1-{Y}^{\left(i\right)})*log(1-{\widehat{y}}^{\left(i\right)})]$$
- Compare the resulting output with the desired output for the defined input using Eq. (15).
  15 $${d Z}^{(i)}={\widehat{y}}^{(i)}- {Y}^{(i)}$$
- Calculate The accumulative weight and bias using Eq. (16-17).
  16 $${dW}_{i}+=\frac{1}{m}({X}_{j}^{\left(i\right)}*d{Z}^{\left(i\right)})$$
  17 $$db+=\frac{1}{m}(d{Z}^{\left(i\right)})$$
- Update the weights $({\text{W}})$ and bias for all neurons using the error function using Eq. (18-19).
  18 $$W_{i} : = ~W_{i} - ~\alpha *dW_{i}$$
  19 $$b: = b - ~\alpha *db$$
- The process is repeated until the error is reduced to an acceptable value which means that the DNN train is successful.

The feature vector is fed directly into the input nodes. These nodes initialized a random number of weights and fine-tuned parameters to the DNN. Each node generates an output using an activation function. The outputs are then connected to the next hidden layers. The activation functions vary across the different hidden layers. Subsequently, the features are retrieved and concatenated to create a new feature vector. The new feature vector is then received by the classifier to determine the confidence of each relation. Then the classifier produces a binary output vector. Training a classifier is the most crucial aspect of the classification process. The role of this phase is to generate a model by training it with a predefined diagnosis class label, which is used later to classify unlabeled diagnoses. The training data is essentially a means of learning the classifier model. In the feed process, after the data has been fed, forward propagation occurs. The losses are compared against the loss function, the parameters are adjusted accordingly based on the incurred losses throughout the training process and patterns that correlate with the desired output, as declared from Eqs. (2)–(18). DNN is trained using a gradient descent algorithm to control the range of the weight values throughout the training phase. We used the ReLU activation function from hidden layers one to nine, as depicted in Figure 6, and a Sigmoid activation function in the last layer as shown in Figure 7. Generally, ReLU and Sigmoid functions are employed for binary classification^21^.

In the Validation process, we run the suggested model on various subsets of both training and validation. This step can be further categorized into two techniques: exhaustive and non-exhaustive cross-validation. In the exhaustive cross-validation approach, training, and testing are performed on all data samples. A portion of the dataset is designated for testing purposes, whereas the remaining portions are used for training. It is also divided into:

- Leave-P-Out Validation: leave p data points from the training data^22^.
- Leave One Out Cross-Validation: the fold count numbers are equal to the total number of dataset samples^22^.

In a non-exhaustive cross-validation approach, the dataset is divided into multiple subsets, each consisting of several blocks. Each block is divided into subsets of training samples and test samples. Therefore, the overall result is the average of all test samples. It is divided into:

- K-fold Cross-Validation involves splitting the data into k subsets. One of the k subsets are used as the validation set, whereas the other k-1 subsets is used as the training set^23^_._
- The holdout method removes a portion of the training dataset and sends it to the model to train the remainder of the dataset^22^.
- Stratified K-fold Cross-Validation works on an imbalanced dataset. Each fold contains approximately the same strata of samples for each output class.

**Figure 9:**
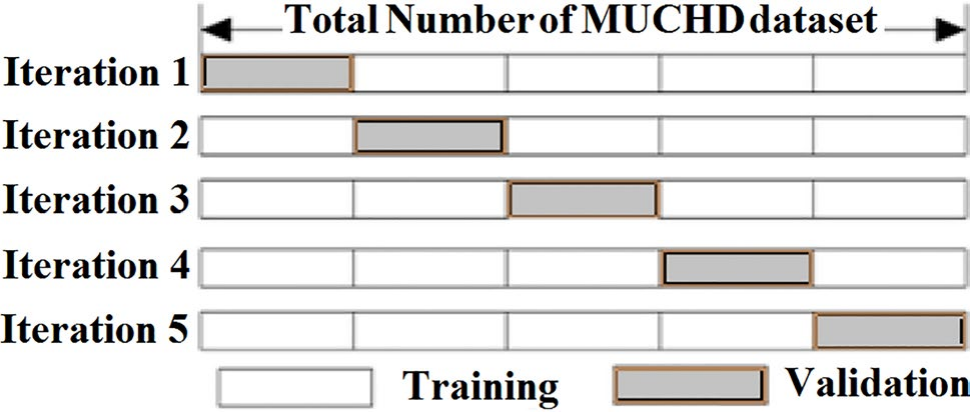
K-fold Validation.

In our model, the data are divided into 5 pieces, each containing 20% of the full dataset portion. We employ K- folds=5, which means that the training portions are 4/5 and only one block is used for validation. In iteration one, we designate the first fold as the validation set and utilize the remaining folds for training. This is valuable for quantitative evaluation to measure model quality based on a 20% holdout set. We repeat this process, using each fold once as the holdout set according to the number of iterations and the averaged error as shown in Figure 9.

**Figure 10:**
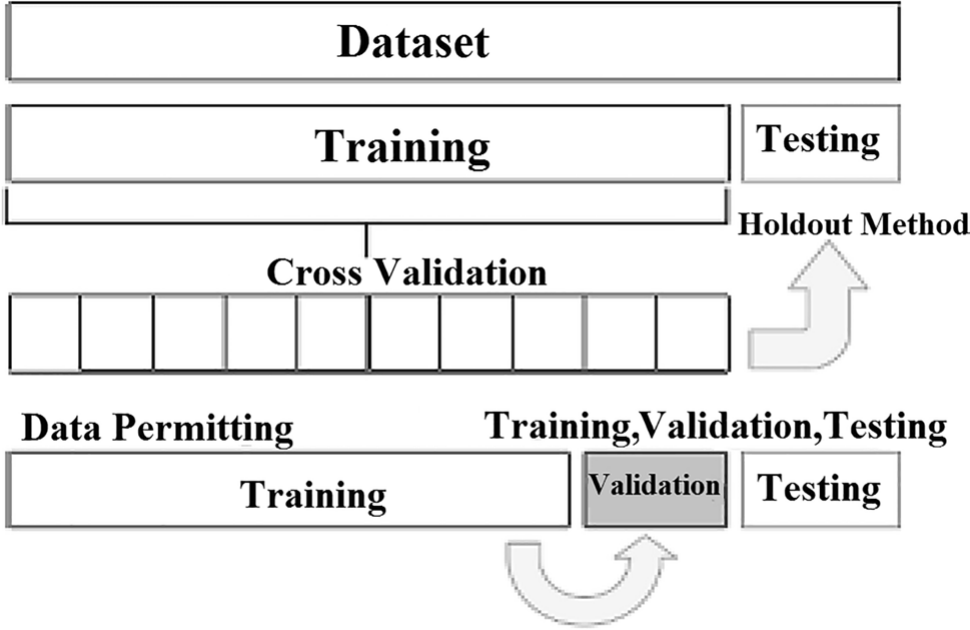
Validation Process.

Stratification is a valuable tool for addressing imbalanced data. This means that if the number of diabetes patients is 75% of class one diabetes, and the non-diabetes patients, comprise 25% of class zero, then the stratification parameter will ensure that the same percentage portion of the data split remains true. The validation structure is presented in Figure 10.

The data with unlabeled classes are prepared previously in the preprocessing phase. The mapping function is employed to classify unlabeled data to determine the label to which they belong. In the test phase, the model finds the data features that correlate with a defined class. The classification technique is then used to assign an accurate class label diagnosis to unlabeled cases, specifically distinguishing between diabetes and non-diabetes disease. The DNN sequential model is trained using various hyperparameters. By experimenting with different values to determine the best-fit parameter such that epochs (the number of training times) have three values (10, 50, 100), batch size (the number of sub-samples fed to DNN after updating the parameter) have six values (10, 20, 40, 60, 80, 100), the optimizer is used to reduce the output error during the backpropagation method. The optimizer is of seven values ('SGD', 'RMSprop', 'Adagrad', 'Adadelta', 'Adam', 'Adamax', 'Nadam'), activation function ('softmax', 'softplus', 'softsign', 'ReLU', 'tanh', 'Sigmoid', 'hard_Sigmoid', 'linear'), initial weights constraint consist of five values (0.01,0.02,0.03,0.04,0.05), neurons are of eleven values (1, 5, 6, 7, 8, 10, 15, 17, 20, 25, 30), momentum has six values (0.0, 0.2, 0.4, 0.6, 0.8, 0.9) and learning rates which control the weight adjustment concerning loss function, gradient function, and outcome. The learning rates are of five values (0.001, 0.01, 0.1, 0.2, 0.3). We use the Keras and Tensor-Flow libraries to create a DNN of sequential models. In DNN, the Stochastic Gradient Descent (SGD) optimizer is required to reduce the output error during the feedforward approach. The train-test split and cross-validation functions from the sci-kit-learn library are used to perform the splitting task.

### Evaluation phase

The evaluation phase allows us to test the model on a validation set to accurately assess its performance in real-world scenarios. The normality assumption states that the difference between the actual output and the predicted output of a model is normally distributed and checked using histograms or a standard normal distribution. We train the proposed DNN model using the updated parameters specific to the MUCHD dataset. We also express the classification loss function by employing binary cross-entropy, which is used to compute the system error see Equation (3.3.6.9).

### Optimizing phase

After completing the evaluation process, there is a high probability that our model can be further optimized using the gradient descent function. We begin by initializing the weights and biases with initial values. Subsequently, we meticulously fine-tuned the backpropagation process and the challenge in training the DNN model lies in the careful selection of the most significant features. Some features may contribute to misleading the result because of noise or null data. To overcome the issue of over-fitting, we fine-tuned the hyperparameters of the model by conducting different experiments only on 70% of the dataset. These parameters encompass the number of hidden layers in the network, the number of neurons, and the activation function used to estimate the output of each neuron. One way to achieve this is to repeatedly utilize the model and increase the number of epochs. The learning rate is a crucial parameter in training the model, with a small positive value within the range between 0.0 and 1.0. A smaller learning rate necessitates more training epochs because it leads to smaller changes in weights during each update. These values have a significant impact on the accuracy of the model and the duration of the training process, particularly complex models, where the most common problems are encountered when a model performs well on the training data but fails to generalize effectively to unseen data. This occurs when the model learns a pattern specific to the training dataset that is irrelevant to other unseen data. There are two ways to avoid overfitting. The first and most effective solution is to acquire more data. However, excessively reducing the network capacity results in underfitting, rendering the model incapable of learning the relevant patterns in the trained data. Unfortunately, there are no magical formulas for determining the ideal balance. This necessitates testing and evaluation by manipulating the number of parameters and observing the subsequent performance. Another way to avoid overfitting is to apply weight regularization to the model. A common method is to assess the complexity of the network and ensure that the weights are constrained to only small values. Regularizing the distribution of weight values involves incorporating a cost into the DNN loss function. This cost comes in two ways. The first is L1 regularization, where the cost is regarded as the absolute value of the weight coefficient. The second is called L2 regularization, which adds cost based on the square value of the weight coefficient.

### Prediction phase

The output of the optimizing phase indicates that the system has been successfully trained and tested. We are now ready to run the classification model for any new data with unknown class labels. This contributes is helpful to obtaining the final recommended classifier for disease prediction. The proposed technique can also be utilized to determine whether an individual has Type 1 diabetes or not. According to the C-Peptide test, the type of diabetes can also be defined easily. If the C-Peptide value is less than 0.2 nmol/l, then the diabetes patient will be Type 1. A high level of C-peptide level can be an indicator of other diabetes types. Table 7 presents the DNN classifier with the corresponding prediction values using the confusion matrix.

**Table 7:** Confusion matrix for the DNN model.

Method		Predicted No	Predicted Yes
DNN model	Actual No	151	0
	Actual Yes	1	396

The DNN technique is executed within the Jupyter Notebook, utilizing the Python language for programming purposes.

### Informed consent

Informed consent was obtained from caregivers of all cases (legal guardians).

### Institutional Research Board (IRP) Approval

The research of this study was approved by the Scientific research ethics of the esteemed Faculty of Computer Science and Information System at Mansoura University, Egypt. The authors confirm that all methods were performed according to the relevant guidelines and regulations by the Faculty of Computer Science and Information System at Mansoura University, Egypt. The approval of IRP was granted on the date of 10/4/2023, and the study has been assigned a unique code number: 202304009 for reference purposes and that the scientific research meets the conditions and ethics of scientific research.

## Results and evaluation

The early detection of diabetes disease is crucial for improving the overall health and well-being of patients. Recently, DNN has been employed to develop accurate prediction models for diabetes. The suggested model is executed using the MUCHD dataset. The dataset is sourced from the Mansoura University Children's Hospital repository. Comprised of 548 samples, each sample contains 18 features including Age, Sex, Duration, Cholesterol, Creatinine, HbA1c, Acetone, Insulin, PCPeptide, FCPeptide, PBGlucose, FBGlucose, RBGlucose, PH, HCO3, Na and K. There are 151 samples labeled as non-diabetes patients (class 0) and 397 samples labeled as diabetes patients (class 1). The supported DNN technique consists of five phases: preprocessing the MUCHD dataset to remove outlier data, normalizing the dataset, replacing missing values with mean values for each feature, training the DNN model with optimized tuning hyperparameters and splitting the dataset into three parts: training, validation, and testing. The training and validation dataset consists of 384 samples, which is 70% of the total, while the remaining 164 samples, equivalent to 30% are reserved for model evaluation and are referred to as the out-of-sample test set. A confusion matrix is presented in Table 8. Patients who have a diabetes disease and are predicted to have a diabetes disease are called True Positives (TP); the patients who are non-diabetic but are predicted to have diabetes disease are called False Positives (FP); patients who are non-diabetic and are predicted to have non-diabetes disease are called True Negatives (TN); and those who have diabetes and are predicted to have non-diabetes disease are called False Negatives (FN) ^24^.

**Table 8:** The Confusion matrix.

	Predicted No (0)	Predicted Yes (1)
Actual No (0)	TN	FP
Actual Yes (1)	FN	TP

TN = True Negative, FP = False Positive, FN = False Negative and TP = True Positive.

**Figure 11:**
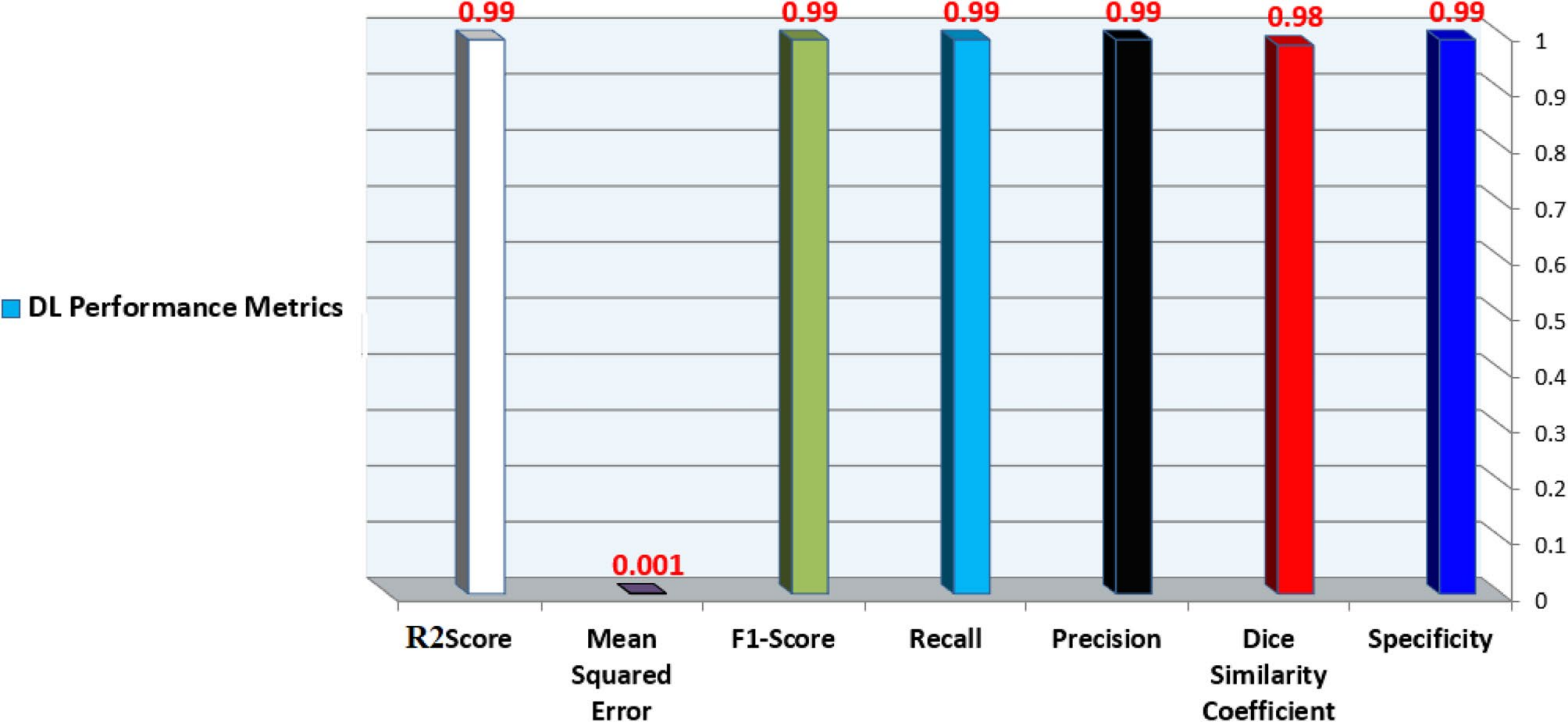
The DNN performance metrics use Precision, Recall, F1-Score, Specificity, Mean Squared Error, and R2 Score.

The classification algorithm is used to evaluate the performance efficiency in terms of Accuracy, Precision, Recall and F1-Score, Training Scores, Mean Square Error, and R2 Score for all samples as shown in Figure 11.

The Specificity, Dice Similarity Coefficient, Precision, Recall, F1-Score, and accuracy performance measures matrices can be calculated from Eqs. (20)–(25)^25^.

- The Specificity asks about how many normal cases are correctly predicted using Eq. (20).
  20 $$Specificity=\frac{TN}{TN+FP}$$
- The Dice Similarity Coefficient determines how many samples are classified correctly using Eq. (21).
  21 $$Dice Similarity Coefficient =\frac{2*TP}{2*TP+FP+FN}$$
- Precision Score provides the accuracy of positive diabetes predictions using Eq. (22).
  22 $$Precision=\frac{TP}{TP+FP}$$
- Recall Score (Sensitivity) is the ratio of correctly predicted positive cases to all samples using Eq. (23).
  23 $$Recall=\frac{TP}{TP+FN}$$
- F1-Score is the weighted average of Precision and Recall using Eq. (24).
  24 $$F1=\frac{2*(Precision*Recall)}{Precision+Recall}$$
- Accuracy indicates DL classifier correctness in the diagnosis process of whether the patient has diabetes or non-diabetes using Eq. (25).
  25 $$Accuracy =\frac{\mathrm{TP }+{\text{TN}}}{\mathrm{TP }+\mathrm{ TN }+{\text{FN}}+\mathrm{ FP}}$$

**Figure 12:**
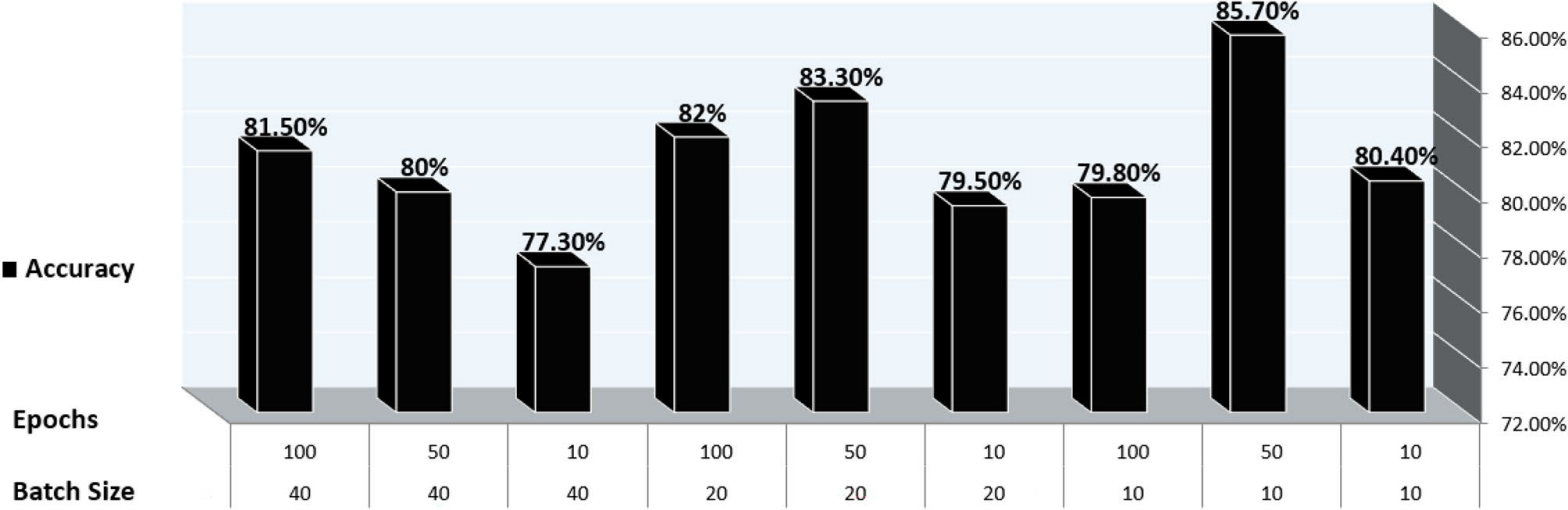
Batch size, Epochs, and accuracy.

**Figure 13:**
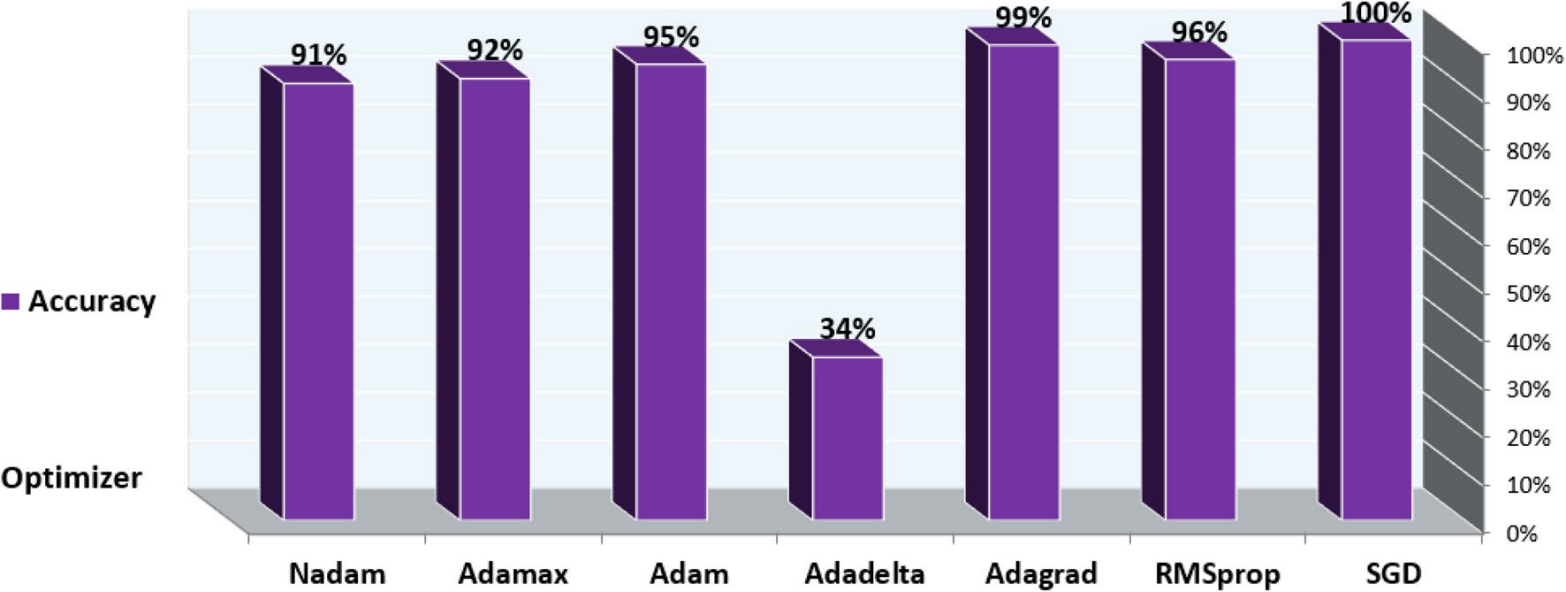
Optimizer and Accuracy.

**Figure 14:**
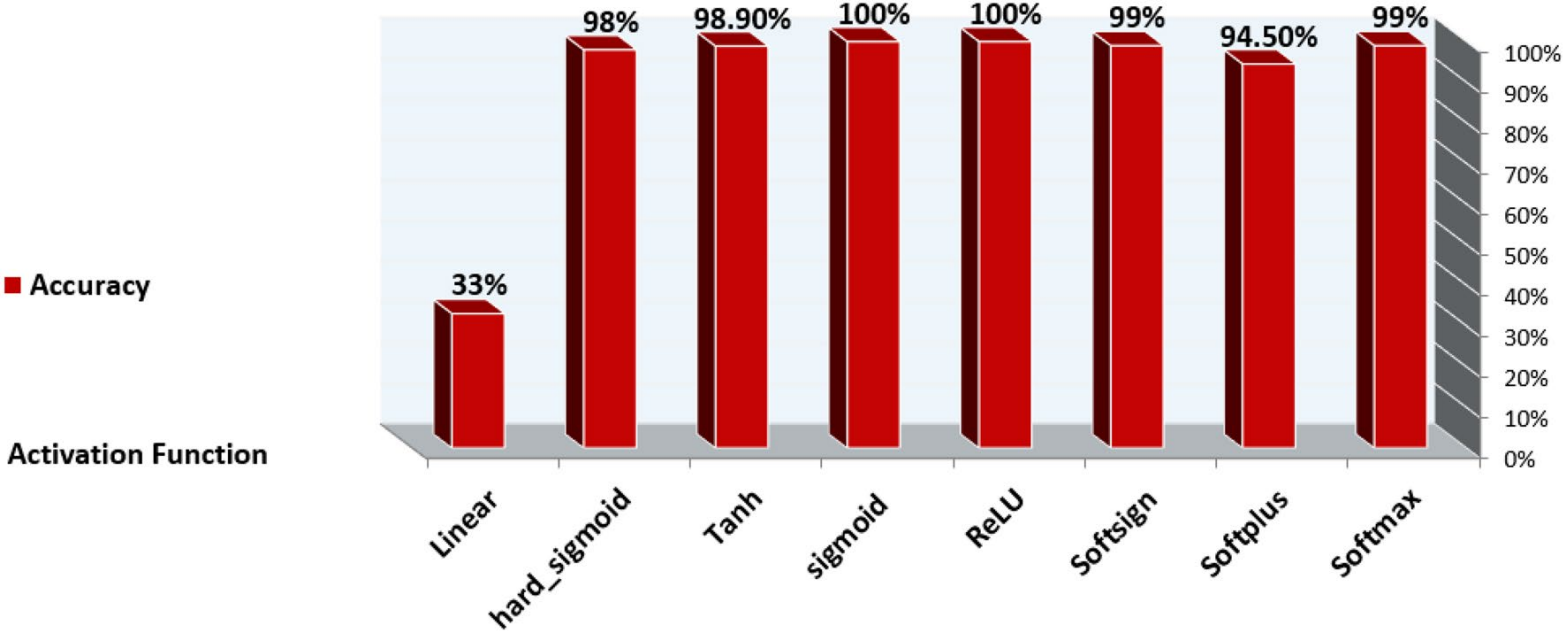
Activation Function and Accuracy.

**Figure 15:**
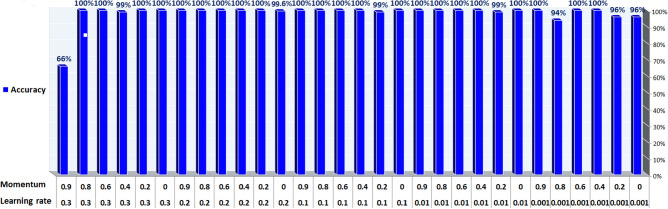
Learning Rate, Momentum, and Accuracy.

Based on the experiments conducted, the hyperparameters configurations are tuned to achieve optimal results with a batch size: 10, 50 epochs, the SGD optimizer, ReLU activation function in all nine hidden layers and Sigmoid activation function in the last hidden layer with a weight constraint of 3, learning rate of 0.01 and momentum of 0.4. The DNN model summary of tuning hyperparameters is reported from Figures 12, 13, 14 and 15.

The proposed system with a DNN classification algorithm is recommended for predicting any other binary disease classification system.

**Figure 16:**
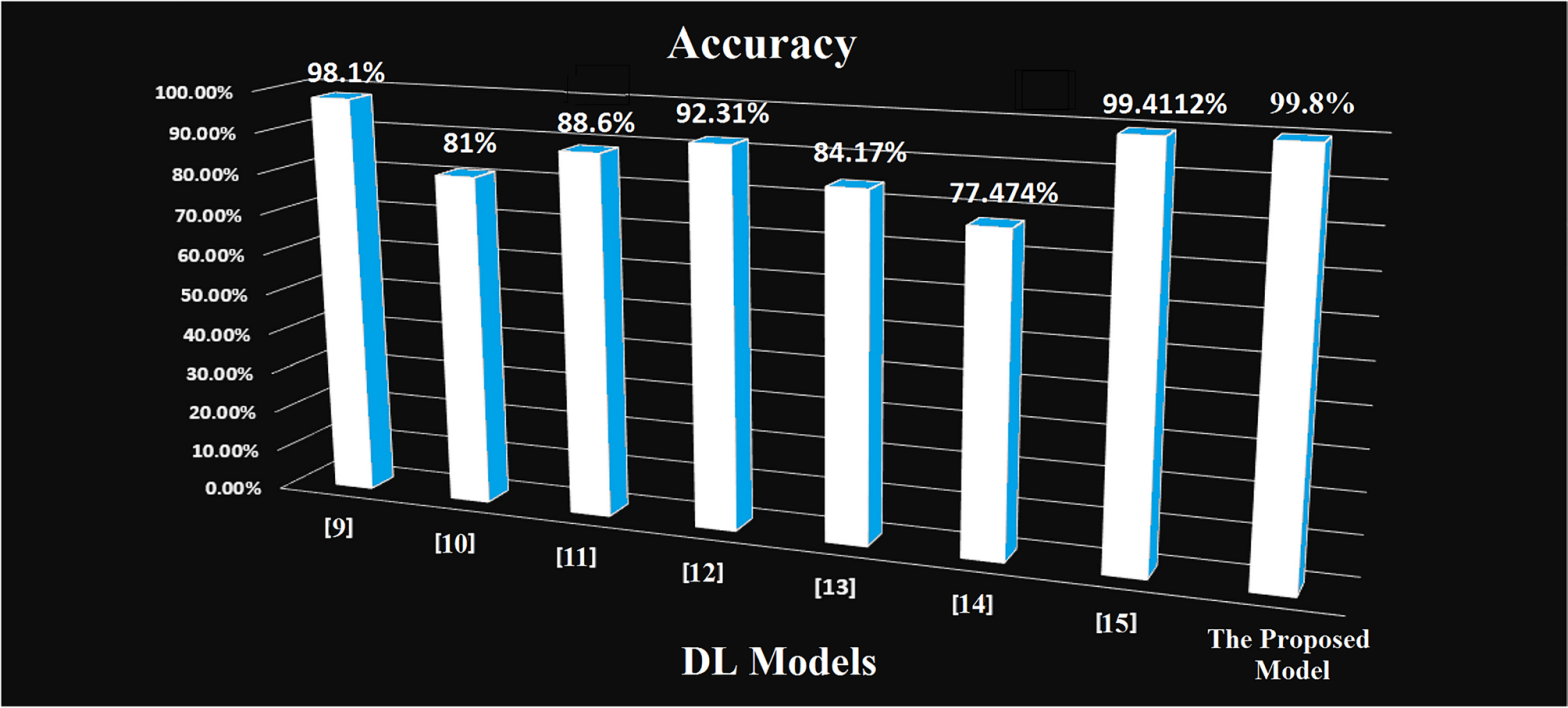
A comparison of accuracy between DL models reviewed and the proposed model.

In this section, we provide a comparison between the proposed system and other methods in the literature, as shown in Figure 16 and from Tables 9 and 10.

**Table 9:** Summarize the performance of the DL models reviewed and the proposed model.

Study	Year	Dataset	Algorithm	Activation Function	Data Preprocessing Technique	Accuracy Metrics
^9^	2023	PID	CNN, DNN, and MLP	Sigmoid or Soft max	- Data and feature augmentation by replacing missing values with the mean value	98.1%
^10^	2022	PID	SVM, LR, ANN, CNN, RNN, LSTM	Sigmoid and RELU	- Replace missing values with the mean value	81%
^11^	2021	PID	DT, NN KNN, RF, NB, AB, LR and SVM	RELU	-WEKA Analysis Tool - Replace the missing values with the mean value - Pearson’s correlation technique	88.6%
^12^	2021	PID	VAE, SAE, MLP and CNN	Sigmoid	- Normalization using Max–Min, Mean, and Logarithmic - Data augmentation using a VAE and feature augmentation using the SAE	92.31%
^13^	2020	PID	MLFFNN	SELU and ELU	- Imputing the missing values with the Mean value	84.17%
^14^	2021	PID	MLP and SVM	Gaussian RBF	Imputing the missing values with the Mean value	77.474%
^15^	2020	PID and DT	DNN	Softmax and linear	Bach normalization using the mean value	99.4112%
The Proposed Model		MUCHD	DNN and MLP	ReLU and Sigmoid	Imputing the missing values with the Mean value	99.8%

**Table 10:** Notations of Table 9 and their descriptions.

Notation	Description
PID	Pima Indian Diabetes Dataset
DT	Diabetes Type Dataset
CNN	Convolutional Neural Network
SVM	Support Vector Machine
LR	Logistic Regression
ANN	Artificial Neural Network
RNN	Recurrent Neural Network
LSTM	Long Short-Term Memory
RBMs	Restricted Boltzmann Machines
DBN	Deep Belief Network
DNN	Deep Neural Network
RELU	Rectified Linear Unit
DT	Decision Tree
KNN	k Nearest neighbors
RF	Random Forest
NB	Naive Bayes
AB	Ada boost
VAE	Variational Auto Encoder
SAE	Sparse Autoencoder
MLP	Multi-layer Perceptron
MLFNN	Multi-Layer Feed Forward, Neural Networks
SELU	Scaled Exponential Linear Unit
ELU	Exponential Linear Unit
Gaussian RBF	Gaussian Radial Basis Function
CPCSSN	Canadian primary care sentinel surveillance network
AI	Artificial Intelligence

Based on these previous studies, the proposed system achieved the best results compared to the others. The advantages of the proposed DNN system are as follows: First, it employs a comprehensive approach to diagnose pediatric diabetes in younger individuals who may struggle to accurately articulate their symptoms. This ensures that no potential signs of disease are observed. Second, the system is versatile and can be applied to different real datasets involving different diseases. This adaptability allows a wider range of medical conditions to be analyzed and effectively diagnosed Third, it saves time, memory, computational cost, and effort by using the pre-trained DNN model. Finally, it is one of the few studies conducted specifically on pediatric patients. In contrast, most of the studies analyzing diabetes applied to adults with a small number of significant features that cannot effectively detect diabetes correctly.

## Conclusion

In this paper, a groundbreaking study was presented for the early detection of diabetes disease. We propose a new model using the DL technique for diabetes disease classification and prediction. The experimental results confirmed the efficiency of the designed system, with an impressive accuracy rate of 99.8%. To evaluate the effectiveness of our model, we conducted a meticulous analysis of a dataset consisting of 548 children using the MUCHD dataset, which encompasses 18 attributes. The proposed system constructed a robust DNN model using the MLP algorithm through a follow-up of phases. In the preprocessing phase, the dataset is cleaned, and the missing values are replaced by the mean value for each input feature of the dataset. The trained model receives these multi-attribute features as input and the train/test phase is presented, followed by the evaluation, optimizing, and prediction phases. Several hyperparameters are fine-tuned to generate an ideal model. We employ two activation functions: ReLU and Sigmoid, which play vital roles in the DNN diabetes prediction model. The model demonstrated good results in various quality measures, including Accuracy, Precision, Recall, F1-Score, Training Score, Mean Squared Error, and R2 Score. These metrics are essential for offering valuable insights into the performance of a model.

The predicted diabetes information can be of great value as a warning signal for both young patients and pediatricians. This aids in making informed decisions and effectively managing diabetes. Additionally, the use of DL techniques can solve feature extraction problems and achieve high success in the binary classification of problem-solving. The Simulation results demonstrate that the proposed model outperforms existing models in terms of accuracy and effectiveness. Furthermore, this system can be applied universally to diagnose different pediatric diabetes symptoms that have a significant impact on the overall well-being and quality of young individuals.

To achieve Further improvement in accuracy, training the model on a larger dataset is recommended. This work can be used to automate diabetes disease prediction with the help of successful machine learning techniques that can be incorporated as a base learner in the proposed framework. Our study can also be extended to determine diabetes types. Furthermore, it is advisable to explore the potential enhancement of the proposed system by combining it with other medical scan images, such as those of the eye, chest, and cardiology, as well as with clinical biomarkers.

## Data Availability

The dataset is not publicly shared due to the imperative need for patient data security. Access to this dataset is strictly limited to authorized individuals who have obtained a clear written agreement from the hospital manager. The data supporting the findings of this study are accessible through the hospital repository system, but only to those who have authorized access. However, it is important to note that restrictions apply to the availability of these data, as they were used under license for the current study and are therefore not publicly available. Should you require access to the data, please submit a reasonable request to the author: Abeer El-Sayed El-Bashbishy, and Email: abeerelbashbishy@gmail.com. However, please be aware that permission from both the hospital manager and the head of the Endocrinology/Diabetes department at Mansoura University Children Hospital is required.
